# Horner Syndrome Secondary to Suspected Internal Carotid Artery Dissection in a Golden Retriever

**DOI:** 10.1111/jvim.70216

**Published:** 2025-08-28

**Authors:** Tommaso Davini, Carlotta Remelli, Chiara Mattei, Swan Specchi, Roberta Biserni, Marco Bernardini

**Affiliations:** ^1^ Anicura “Ospedale Veterinario I Portoni Rossi” Zola Predosa Bologna Italy; ^2^ Antech Imaging Service Fountain Valley California USA; ^3^ Department of Animal Medicine, Production and Health University of Padua Legnaro Padua Italy

**Keywords:** anisocoria, canine, computed tomography angiography, magnetic resonance angiography

## Abstract

A 5‐year‐old male golden retriever was presented after a subacute onset of left‐sided Horner syndrome (HS). The dog had anisocoria with left‐sided miosis, ptosis of the upper eyelid, and third eyelid protrusion in the left eye. Because of the absence of additional neurological abnormalities, clinical signs were suggestive of left isolated HS, and the lesion was localized at the level of either the preganglionic or postganglionic neuron of the sympathetic chain. Magnetic resonance imaging (MRI) of the head and total body computed tomography (CT) identified marked narrowing and irregularity of the left internal carotid artery (ICA) in addition to loss of normal vessel flow‐void and T1‐weighted hyperintensity in the lumen of the left ICA. Except for these abnormalities, MRI and CT results were normal. These findings were suggestive of left internal carotid artery dissection (ICAD), suggesting that ICAD should be considered as a possible differential diagnosis of HS in dogs.

**Abbreviations**
CeAD: cervical artery dissection
CT: computed tomography
CTA: computed tomography angiography
DWI: diffusion‐weighted imaging
FATSAT: fat‐saturated
FLAIR: fluid‐attenuated inversion recovery
FSE: fast spin echo
HS: Horner syndrome
ICA: internal carotid artery
ICAD: internal carotid artery dissection
MRI: magnetic resonance imaging
SWI: susceptibility‐weight images
T1W: T1‐weighted
T2W: T2‐weighted
TOF‐3D MRA: 3D time‐of‐flight magnetic resonance angiography

## 1 Introduction

Horner syndrome (HS) is a clinical condition well described in human and veterinary medicine, which is caused by disruption of the sympathetic innervation to the eye [1]. In dogs and cats, it is characterized by miosis, upper eyelid ptosis, enophthalmos, and third eyelid protrusion [1, 2]. Based on the position of the lesion, HS is classified as postganglionic, preganglionic, or central [2]. Many conditions can cause HS in dogs, such as iatrogenic, neoplastic, traumatic, and infectious diseases [1, 2]. However, a cause frequently is not identified, and idiopathic HS represents approximately half of the cases in dogs [3, 4, 5]. Studies have reported a high prevalence of idiopathic HS in male golden retrievers [6, 7, 8, 9].

In human medicine, the differential diagnosis for HS includes internal carotid artery dissection (ICAD) [10]. This condition is the most commonly identified underlying cause for isolated HS (i.e., HS that is not accompanied by additional neurological deficits in humans) [11].

To our knowledge, ICAD has not been described as a cause of HS in dogs. This case report presents the clinical and imaging features of HS suspected of being related to ICAD in a golden retriever.

## 2 Case Description

**FIGURE 1:**
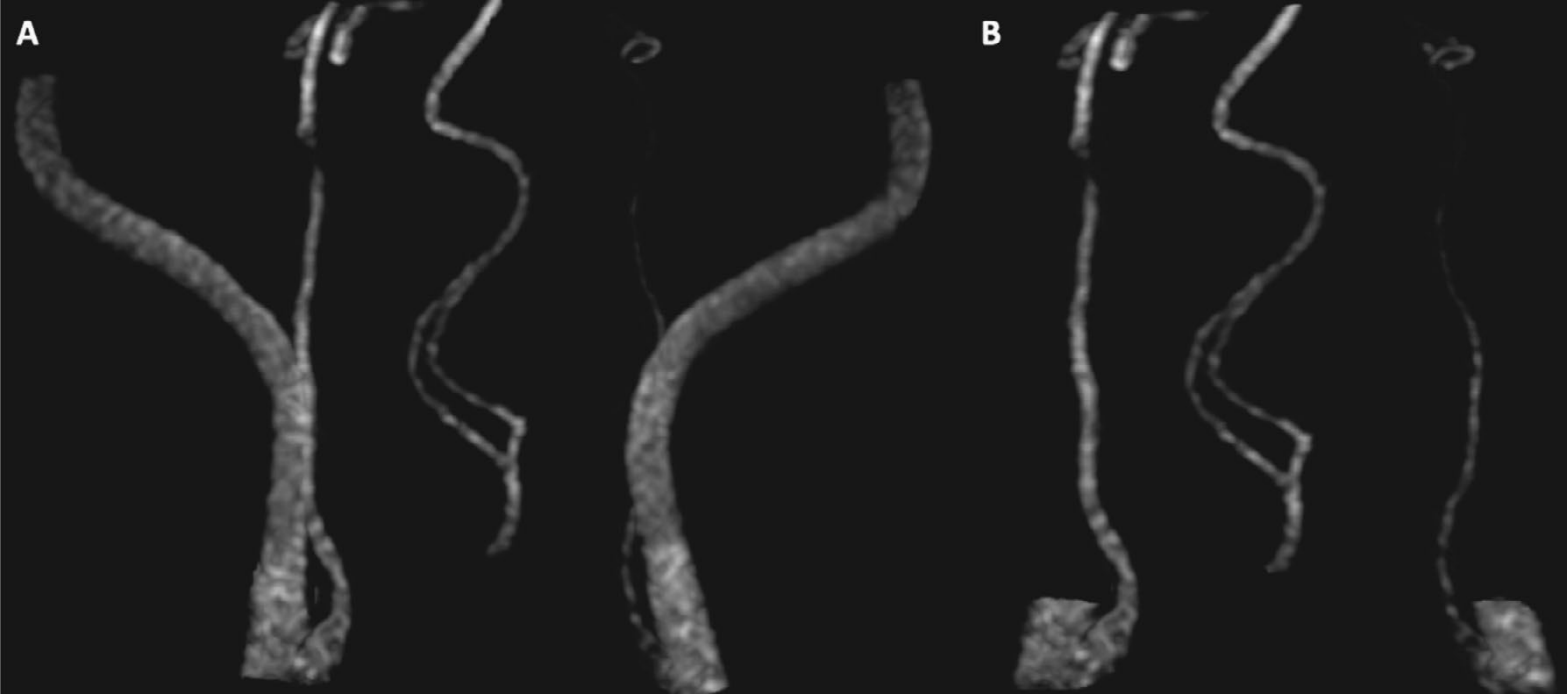
3D time‐of‐flight magnetic resonance angiography in maximum intensity projection reconstructed before (A) and after (B) decoupage, including both internal carotid arteries (ICAs) and the basilar artery in the center, viewed in the dorsal plane, showing progressive narrowing of the left ICA.

**FIGURE 2:**
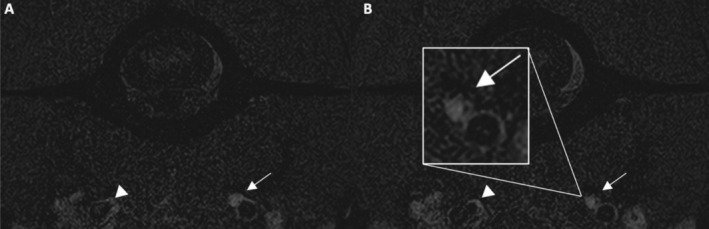
Transverse fast spin echo T1W fat‐saturated at the level of the atlas. There is loss of flow‐void along with hyperintensity into two consecutive slices (A, B), in the left internal carotid artery after its origin from the common carotid artery (white arrow), compared to the contralateral vessel (white arrowhead), consistent with a thrombosed false channel. Box (B) shows enlargement of the above mentioned abnormalities.

**FIGURE 3:**
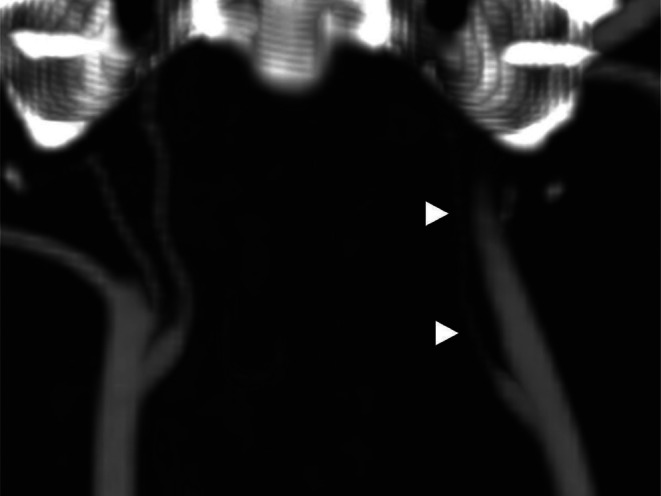
Remarkable left‐sided internal carotid artery lumen diameter reduction and irregularity on dorsal computed tomography angiography maximum intensity projection image (white arrowheads).

A 5‐year‐old male golden retriever was presented with an acute onset of upper eyelid ptosis and third eyelid protrusion in the left eye. Other than these abnormalities, the dog was reported to be clinically normal. The dog had received initial medical treatment that included topical antibiotics and anti‐inflammatory medications. Because of a lack of improvement after 10 days, the dog underwent further investigation. A general clinical examination, including fundus examination, was normal. The CBC and serum biochemistry profile were within normal limits. Neurologic examination disclosed anisocoria with left‐sided miosis, ptosis of the upper eyelid, and third eyelid protrusion in the left eye. The remainder of the neurologic examination was normal. Neurologic deficits were suggestive of left‐sided isolated HS. The lesion was localized at the level of either the preganglionic or postganglionic neuron of the sympathetic chain. Because of the absence of other neurological signs and breed predisposition, the main differential diagnosis was idiopathic HS. Neoplastic and infectious or inflammatory conditions were considered less likely. The dog underwent magnetic resonance imaging (MRI) of the head and total body computed tomography (CT). The MRI of the head was acquired under general anesthesia with a high‐field MRI scanner (1.5 Tesla Vantage Elan, Canon Medical Systems Europe B.V., Netherlands). Fast spin echo (FSE) T2‐weighted (T2W) sequences were acquired in the sagittal plane. The FSE T2W, FSE T1‐weighted (T1W) sequences pre‐ and post‐IV contrast administration (0.2 mL/kg gadoteric acid), FSE T1W fat‐saturated (FATSAT), fluid‐attenuated inversion recovery (FLAIR), diffusion‐weighted imaging (DWI), and three‐dimensional (3D) time‐of‐flight (TOF‐3D) magnetic resonance angiography (MRA) sequences were acquired in the transverse plane. Susceptibility‐weighted images (SWI) in the dorsal plane also were acquired. Magnetic resonance imaging of the head was unremarkable except for TOF‐3D MRA and FSE T1W FATSAT. The TOF‐3D MRA showed progressive luminal narrowing of the left internal carotid artery (ICA) soon after its origin from the common carotid artery, followed by a filiform aspect over its course (Figure 1). The FSE T1W FATSAT transverse images indicated loss of normal vessel flow‐void along with T1W hyperintensity in almost the entire lumen of the left ICA immediately cranial to the bifurcation of the common carotid artery (Figure 2). Total body CT, performed under general anesthesia, using a 128‐slice CT scanner (Somatom Perspective, Siemens Healthineers) with 600 mg/kg IV of the iodinated nonionic contrast medium iopamidol (Iopamiro 300 mg/mL, Bracco Imaging S.p.A.) was normal, except for the left ICA having marked narrowing and irregularity along its course; this narrowing was more pronounced above the carotid bifurcation and highlighted in all post‐contrast sequences (Figure 3). Based on clinical presentation and diagnostic findings, a presumptive diagnosis of left ICAD was made.

## 3 Discussion

Sympathetic innervation to the eye is a three‐neuron system comprising both upper and lower motor neurons, the pathway of which has been extensively described in veterinary literature [1, 2, 12, 13]. Briefly, it begins in the hypothalamus and proceeds caudally within the lateral tectotegmentospinal system in the brainstem and in the lateral funiculus of the spinal cord, terminating in the intermediolateral gray horn of the first, second, and third thoracic spinal cord segments. Here, the axons of the first‐order neurons synapse with the second‐order (preganglionic) neuronal cell bodies. Their axons exit the spinal cord, joining the ventral nerve roots and the proximal portion of the spinal nerves as far as where they branch to join the thoracic sympathetic trunk. After passing through the cervicothoracic and middle cervical ganglia, these axons course cranially in the cervical sympathetic trunk in association with the vagus nerve to form the vagosympathetic trunk, located in the carotid sheath. After separating from the vagus nerve, the cervical sympathetic trunk synapses in the cranial cervical ganglion. The axons of the third‐order (postganglionic) neuron pass through the tympano‐occipital fissure and travel via the petrous temporal bone to exit the cranial cavity as a component of the ophthalmic branch of the trigeminal nerve, which enters the orbit to innervate the orbital muscles, the eyelids (including the third eyelid), and the dilator pupillae. Any lesion along this pathway can result in HS [9].

Horner syndrome describes characteristic clinical signs of major clinical relevance because they indicate that the oculosympathetic pathway has been interrupted [6]. In the absence of other concomitant neurological deficits that could facilitate neurolocalization, clinical signs will be the same regardless of the location of the lesion [2]. In cases defined as isolated HS, it is of crucial importance to perform pharmacological tests to identify the affected part of the sympathetic innervation of the eye to list appropriate differential diagnoses [2]. In both veterinary and human medicine, many underlying causes are described. In veterinary medicine, isolated HS is most likely to be idiopathic, with golden retrievers overrepresented, and the etiopathogenesis currently remains a topic of discussion [7, 8, 9]. In human medicine, some authors propose an investigative algorithm applicable in clinical settings [10], especially in patients with localizing clinical signs. Nevertheless, quite commonly, human patients present with isolated HS. Limited availability of specific pharmacological agents during acute assessment and denervation hypersensitivity or transmitter depletion, which may not have occurred already in the acute phase, makes approaching these patients a diagnostic dilemma, especially in urgent settings [10]. Different authors have investigated the presence of a causative lesion in patients with isolated HS and reported that 20%–54% of human patients have a causative lesion on imaging, with the most common finding represented by ICAD [11, 14]. Cervical artery dissection (CeAD) may affect the vertebral arteries, the ICA or both, the latter being considered the most commonly affected site for cervical dissection [15]. From a pathophysiological point of view, the most common trigger for CeADs is a tear in the tunica intima of the arterial wall, predisposing it to the formation of a false lumen, which causes an intramural hematoma [16]. This, in turn, leads to arterial stenosis [16]. Cervical artery dissections originate spontaneously in most cases, even if different causes, such as traumatic events, connective tissue disorders, infectious diseases, hypertension, and anatomical abnormalities (e.g., impingement of ICA caused by an elongated styloid process) are described [15, 16]. Clinical implications frequently reported in people are ischemic stroke, head and neck pain, HS, and cranial neuropathies caused by local compression of cranial nerves IX, X, XI, and XII [16].

In humans, axons from third‐order neurons travel as a plexus in the sheath of the ICA [17]. For this reason, in ICAD, the false lumen also can stretch the adjacent sympathetic plexus, leading to postganglionic HS [16, 17]. In veterinary medicine, the path of the postganglionic axons is not well defined, but the microscopic finding of unmyelinated axons coursing with the ICA in the carotid canal supports a similar pathway to that reported in humans [12]. Magnetic resonance imaging is considered the modality of choice for the diagnosis of ICAD in people [15]. Characteristic imaging findings are partial or complete loss of normal vessel flow void on T1W and T2W images [18]. Because of the presence of intracellular and extracellular methemoglobin, the false lumen or intramural thrombus appears as a crescentic hyperintensity on T1W MR images in the subacute phase, which is best appreciated when fat‐saturation is applied [16, 17, 18, 19]. With regard to the arterial lumen, MRA will identify both segmentary stenosis and progressive narrowing leading to occlusion [19]. Noncontrast CT frequently is normal in these cases [19, 20]. Computed tomography angiography (CTA) can identify pseudoaneurysm formation or vessel narrowing. In this regard, CTA can show the same abnormalities of the lumen's vessel diameter identified by conventional angiography, known as the angiographic string sign, which refers to the thin string of intravascular contrast distal to a stenotic site in the ICA [15, 19, 20].

Our case, a male golden retriever with left isolated HS, would have been classified as having idiopathic HS according to a conventional diagnostic evaluation based on standard imaging. Recognition of progressive luminal narrowing of the left ICA on TOF‐3D MRA, along with the almost complete loss of vessel flow void associated with hyperintensity on FSE T1W FATSAT, closely resembles the specific MRI abnormalities reported in human medicine in patients with subacute ICA dissection. Moreover, the findings identified on post‐contrast CT at the level of the left ICA support this diagnosis. The combination of clinical signs, exclusion of any other involvement affecting the remainder of the entire ipsilateral oculosympathetic pathway on both MRI and CT scans, and the recognition of the abovementioned MRI and CT abnormalities support the presence of presumptive left ICAD in this patient.

Experimental studies have described models of carotid artery dissection in dogs and pigs [21, 22], but no ICAD has been described in dogs with isolated HS. Horner syndrome is a possible immediate postoperative and short‐ and long‐term complication detected in dogs undergoing surgery for carotid body paraganglioma, an extra‐adrenal neuroendocrine neoplasm arising at the bifurcation of the common carotid artery [23]. In addition, HS was described as a complication in 7 of 16 cats after carotid artery catheterization [24]. Together, these findings support the presence of third‐order sympathetic axons traveling in or near the carotid sheath of the ICA in dogs and cats, supporting the clinical relevance of the ipsilateral ICA MRI abnormalities described in our case.

The diagnostic evaluation was incomplete in our case, in that it lacked pharmacological testing. In veterinary medicine, the application of direct sympathomimetic drugs (e.g., 0.1% or 1% phenylephrine) commonly is used to localize HS within the oculosympathetic system, based on the principle of denervation hypersensitivity [1, 2]. However, because this phenomenon can take up to 3 weeks to develop, using this test before this period of time has elapsed may incorrectly localize the lesion [2]. Although the accuracy of this test has been questioned [1], pharmacological confirmation of a lesion localized at a postganglionic level could have strengthened our presumptive diagnosis, but the absence of any detectable abnormalities in the entire oculosympathetic pathway on both CT and MRI decreases the likelihood that the changes detected on ICA represented an incidental finding.

## 4 Conclusion

Magnetic resonance imaging and CT abnormalities of the left ICA identified in our patient closely resemble the characteristic imaging findings reported in humans with ICAD, making it likely that left isolated HS was secondary to ipsilateral ICAD. Magnetic resonance angiography, which is considered the gold standard for diagnosis of this pathology in human medicine, is still not routinely performed in veterinary medicine. This factor, in addition to the lack of a standardized investigative algorithm for veterinary patients affected by HS, may have contributed to the underestimation of the prevalence of ICAD in dogs to date. Our results suggest that ICAD should be added to the differential diagnosis of HS in dogs, and the addition of MRA sequences in the MRI protocol for dogs affected by isolated HS should be considered.

## Disclosure

Authors declare no off‐label use of antimicrobials.

## Ethics Statement

Authors declare no institutional animal care and use committee or other approval was needed. Authors declare human ethics approval was not needed.

## Conflicts of Interest

The authors declare no conflicts of interest.
